# Human Genomic Deletions Generated by SVA-Associated Events

**DOI:** 10.1155/2012/807270

**Published:** 2012-05-20

**Authors:** Jungnam Lee, Jungsu Ha, Seung-Yeol Son, Kyudong Han

**Affiliations:** ^1^Department of Nanobiomedical Science and WCU Research Center, Dankook University, Cheonan 330-714, Republic of Korea; ^2^Department of Microbiology, College of Advance Science, Dankook University, Cheonan 330-714, Republic of Korea

## Abstract

Mobile elements are responsible for half of the human genome. Among the elements, L1 and *Alu* are most ubiquitous. They use L1 enzymatic machinery to move in their host genomes. A significant amount of research has been conducted about these two elements. The results showed that these two elements have played important roles in generating genomic variations between human and chimpanzee lineages and even within a species, through various mechanisms. SVA elements are a third type of mobile element which uses the L1 enzymatic machinery to propagate in the human genome but has not been studied much relative to the other elements. Here, we attempt the first identification of the human genomic deletions caused by SVA elements, through the comparison of human and chimpanzee genome sequences. We identified 13 SVA recombination-associated deletions (SRADs) and 13 SVA insertion-mediated deletions (SIMDs) in the human genome and characterized them, focusing on deletion size and the mechanisms causing the events. The results showed that the SRADs and SIMDs have deleted 15,752 and 30,785 bp, respectively, in the human genome since the divergence of human and chimpanzee and that SRADs were caused by two different mechanisms, nonhomologous end joining and nonallelic homologous recombination.

## 1. Introduction

Human diverged from chimpanzee, its most closely related species, ~six million years ago [1, 2]. Since then, the human genome has evolved independently from the chimpanzee genome, leading to human-specific insertions and deletions (INDELs). Species-specific insertion of mobile elements and subsequent genomic rearrangements are major factors causing the INDELs between human and chimpanzee genomes [3–9]. The mobile elements account for ~45% of the human genome. Among them are L1 and *Alu* elements, which are the most successful non-LTR (long terminal repeat) retrotransposon families in the human genome, comprising 13% and 17% of the genome, respectively [10]. A third family of retrotransposon is the SVA element, which is a composite repetitive element, composed of SINE, VNTR, and *Alu*. SVA elements are currently active in the human genome and are involved in the creation of novel primate genes and the development of human disease through various mechanisms including exon-trapping and 5′ transduction [9, 11–14]. The commonality of the three elements is that they use L1 enzymatic machinery to retrotranspose in their host genomes. They mobilize via a “copy and paste” mechanism; they transcribe their RNA intermediates, and the RNA intermediates integrate into new genomic regions in a process known as retrotransposition. The mechanism for this process is called target-primed reverse transcription (TPRT) [15, 16].

L1 and *Alu* have caused INDELs between the human and chimpanzee genomes through various mechanisms such as L1 insertion-mediated deletion [6], *Alu* recombination-mediated deletion [5, 7], and L1 recombination-associated deletion [4]. Genomic rearrangements that are mediated by L1 and *Alu* elements have been widely studied [3–8]. However, the genomic modifications caused by the SVA elements have not been studied much. A total of 2,762 SVA elements have been identified in the human genome [9]. Compared with the L1 and *Alu* elements, a very small number of SVA elements exist in the current human genome. The evolutionary history of the three mobile elements could explain why the number of SVA elements is much lower than that of other elements. The SVA element is the youngest retrotransposon family in primates and, thus, has had less time to propagate; the SVA element emerged in the primate genome after the split between the Old World monkeys and apes, ~20 million years ago (mya) [9]. In contrast, L1 and *Alu* emerged in the mammalian genome ~100 mya [17] and in the primate genome ~60 mya [18], respectively. However, we are unable to estimate how many mobile elements will retrotranspose in the human genome during a certain time because they have been inserted into their host genome at irregular retrotransposition rates: L1 elements presented high activity just after the divergence of the Old World and New World monkeys that occurred approximately 40 mya [19] and *Alu* elements showed high retrotransposition rates 60 to 35 mya [20]. These two time zones are far earlier than the time when the SVA elements emerged in the primate genome. However, SVA elements have been shown to be currently retrotransposoning in the human genome [9] and have the potential to rearrange the human genome.

It has been considered that the SVA elements are one of the factors modifying the human genome through various mechanisms mentioned above. However, an SVA whole genome analysis, except for SVA insertion in the human genome, has not been conducted. In this study, we carried out whole genome analysis of SVA-associated events, SRADs and SIMDs, in the human. We identified 13 SRADs and 13 SIMDs and confirmed the SRAD loci using polymerase chain reaction (PCR) display. Among the SRADs, one was an SVA recombination-mediated deletion. This study is the first whole genome analysis investigating the human genomic deletion generated by SVA-associated events and shows the possibility that SVA elements in the human genome are candidate factors able to cause genomic rearrangements and subsequently genomic variation in human populations.

## 2. Materials and Methods

### 2.1. Computational Data Mining and Manual Inspection

To identify SRADs in the human genome, we first extracted all SVA elements from the genome, based on the UCSC Table Browser utility (http://www.genome.ucsc.edu/cgi-bin/hgTables?org=Human&db=hg19&hgsid=226995881&hgta_doMainPage=1/). For each SVA locus, we extracted 1 kb of the flanking sequence in either direction of the genomic position and used the human sequence as a query to search against the chimpanzee genome sequence using UCSC's BLAT utility (http://www.genome.ucsc.edu/cgi-bin/hgBlat/). We utilized the February 2009 freeze of the human (hg19) genome and the October 2010 freeze of the chimpanzee (panTro3) genome from the UCSC to compare the human and chimpanzee genome reference sequences. For each hit in the BLAT search, we retrieved the human and chimpanzee sequences and annotated repeat elements existing in the sequences, utilizing RepeatMasker (http://www.repeatmasker.org/cgi-bin/WEBRepeatMasker/) analysis. In the case of a candidate SRAD event, a part of the SVA element and its flanking sequence found in the chimpanzee sequence were deleted in the orthologous human locus.

To identify SIMDs, we retrieved human-specific SVA elements. For each SVA locus, we extracted 2 kb flanking sequence in either direction of the element and used the human sequence as a query to search against the chimpanzee genome sequence using UCSC's BLAT utility. For each hit in the BLAT search, we extracted the human and chimpanzee sequences and annotated repeat elements existing in the sequences, utilizing RepeatMasker analysis. In the case of a candidate SIMD event, chimpanzee sequence contained extra sequences compared with the orthologous human sequence.

### 2.2. PCR Amplification and DNA Sequence Analysis

After the manual inspection, we verified the candidate deletions by PCR assay with four different DNA templates: *Homo sapiens *(human), *Pan troglodytes* (common chimpanzee), *Gorilla gorilla* (gorilla), and *Pongo pygmaeus* (Bornean orangutan). Oligonucleotide primers for amplification of SRAD loci were mostly designed using the software Primer3 (http://frodo.wi.mit.edu/primer3/). Then, we used OligoCalc [21] to verify whether the designed primers were self-complimentary and UCSC's *insilico* PCR utility (http://www.genome.ucsc.edu/cgi-bin/hgPcr?command=start/) to computationally test the efficiency of the primers. In case where the primers designed by Primer3 were not desirable for actual PCR amplification, we manually aligned the human, chimpanzee, and orangutan DNA sequences using the software BioEdit v.7.1.3 [22] and designed alternative primers on the conserved region among the DNA sequences. The sequences of the oligonucleotide primers, annealing temperatures, and PCR product sizes are shown in (Supplementary Table 1 in Supplementary Materials available online at doi:10.1155/2012/807270). PCR amplification of each candidate was performed in 20 *μ*L reaction using 10–50 ng template DNA, 200 nM of each oligonucleotide primer, and 10 *μ*L of EmeraldAmp GT PCR Master Mix (Takara, Ohtsu, Japan). Each sample was subjected to an initial denaturation step of 5 min at 95°C, followed by 35 cycles of PCR for 30 sec of denaturation at 95°C, 30 sec at the annealing temperature, 1 to 2 min of extension at 72°C, depending on the product sizes, followed by a final extension step of 10 min at 72°C. For the case where the expected product size was >2 kb, we used Ex Taq polymerase (Takara, Ohtsu, Japan), KOD (Toyobo, Osaka, Japan), and 2X EF-Taq Pre mix 2 (SolGent, Seoul, Republic of Korea) to carry out PCR in 40 *μ*L reactions following the manufacturer's protocol. The resulting products were loaded on 1% agarose gels, stained with ethidium bromide, and visualized using UV fluorescence (Bio-Rad, Hercules, CA, USA). In case where the chimpanzee reference genome sequence contains unsequenced region (Ns) on candidate deletion locus, we sequenced chimpanzee PCR product amplified from the locus to determine the precise deletion size in the human genome. The chimpanzee PCR product was purified from the PCR solution using the Wizard SV Gel and PCR Clean-Up System (Promega, Madison, WI, USA) and cloned into vector using the MG TOPcloner TA Core Kit (Macrogen, Seoul, Republic of Korea), according to the manufacturer's instructions. The sequencing of the colony PCR product was conducted using dideoxy chain-termination sequencing at Macrogen. The DNA sequence from this study has been deposited in Genbank under accession number JQ354986.

### 2.3. Analysis of Flanking Sequences of SVA-Associated Deletions (SADs)

For flanking sequence GC content analysis, we used in-house Perl scripts to extract 10 kb of flanking sequence in either direction of each SAD locus and to calculate the percentage of GC nucleotides in the combined 20 kb of the flanking sequence. To determine whether each SAD event occurs in either intergenic or intragenic region, we used UCSC's Genome Browser utility (http://www.genome.ucsc.edu/cgi-bin/hgGateway?org=Human&db=hg19&hgsid=244865559/).

## 3. Results and Discussion

### 3.1. Whole Genome Analysis of SAD Events

**Figure 1:**
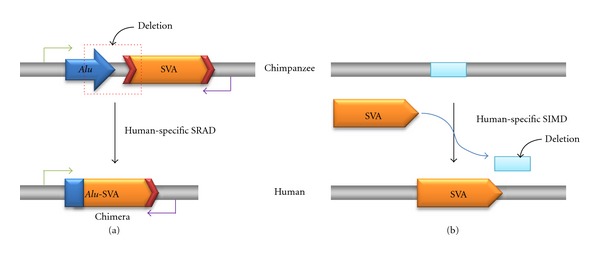
SRAD and SIMD in the human genome. This figure shows the mechanisms underlying SRAD and SIMD in the human genome. (a) SRAD event. In the illustration of the chimpanzee, *Alu* and SVA elements are intact but only one chimeric element exists in the illustration of human-specific SRAD. For both illustrations, two arrows indicate the positions where each PCR primer anneals. (b) SIMD event. This illustration depicts the insertion of the SVA element and the deletion of genomic DNA (blue box).

We first extracted 3,608 SVA elements from the human genome and then excluded 2,206 human-specific SVA elements from the data to identify potential SRAD events in the human genome. However, the copy number of SVA elements seems to be overestimated because some of the SVA elements contained duplications of the VNTR region, and each of those elements was counted as two separate elements instead of one by RepeatMasker utility. We further examined the remaining 1,402 SVA elements, which are shared between human and chimpanzee genomes, to determine whether the elements were associated with deletions in the human genome. We identified 13 SRADs in the human genome by comparing the human and chimpanzee sequences for each locus. They seemed to occur in a low frequency compared to 73 L1 recombination-associated deletions in the human genome [4]. However, for the comparison, we need to consider their copy numbers: ~3,000 SVAs versus ~520,000 L1s in the human genome [9, 10]. We were not computationally able to verify the authenticity of the SRADs because most of the deletions involved portions of the SVA elements which are not shared between human and gorilla genomes. As shown in Figure 1(a), we experimentally verified the authenticity of the SRAD loci by wet-bench PCR analysis. However, we could not experimentally confirm two of the loci because they contained a high density of repetitive elements. Repetitive elements inhibit PCR amplification of their respective genomic regions. To identify potential SIMD events, we retrieved the 2,206 human-specific SVA elements and manually inspected them by the strategy described in Section 2. Through the process, we identified 13 SIMDs in the human genome. Unlike SRADs, we were able to verify the SIMDs (Figure 1(b)) and determine their deletion sizes using the chimpanzee (panTro3), gorilla (gorGor3), and orangutan (ponAbe2) reference genome sequences.

**Figure 2:**
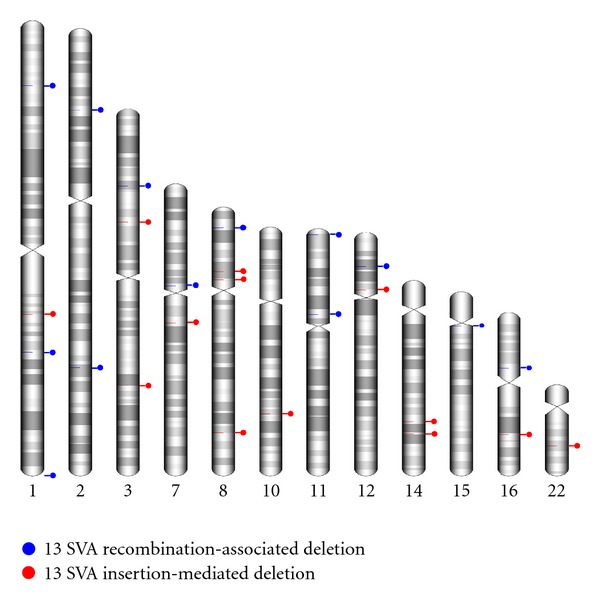
The 28 SVA-associated deletion loci in the human genome. Blue and red circles indicate SRAD and SIMD events, respectively. The karyotype images were created by using the idiographica webtool [23].

The genomic positions of the SRAD and SIMD loci are described in Tables 1 and 2, respectively. In addition, their locations along human chromosomes are shown in Figure 2. Three of the SRAD loci were located on chromosome one. A previous study about SVA elements reported that the observed copy number of SVA elements is much higher than the expected copy number on human chromosome one [9].

### 3.2. Mechanisms Causing SRADs

**Figure 3:**
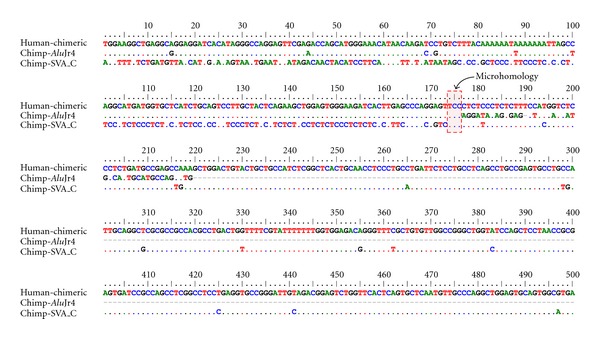
Sequence alignment identifying the microhomology involved in an NHEJ-SRAD event. The chimeric element in human and the *Alu* and SVA elements in chimpanzee are shown. Each dot indicates an identical nucleotide among the elements. Otherwise, differences among the elements are shown with letters. The microhomology for this event is shown in the red box.

We closely examined the loci to elucidate mechanisms that caused the deletions. The results showed that 12 SRADs occurred by nonhomologous end joining (NHEJ) and only one locus occurred by nonallelic homologous recombination (NAHR) between poly-A tails of two different SVA elements (Table 1). NHEJ typically involves microhomology between sequences, and Figure 3 shows the microhomology of our elements [24–26]. It was suggested that NHEJ is one of the repair mechanisms for double-strand breaks (DSBs). Thus, we suspect that SVA element involved in an NHEJ-SRAD event was present in the flanking region of DSBs and microhomology between SVA and the other elements is associated with end binding to bridge the DNA lesion. However, this mechanism is not specific to SVA elements, and other mobile elements including L1 would act in the same way as SVA [4]. At an NAHR-SRAD locus, the two closely related prerecombination SVAs found in the chimpanzee genome are seen to have recombined into a single chimeric SVA in the human genome, having resulted in the deletion of a portion of each SVA element and the intervening DNA sequences. Compared with L1 or *Alu* recombination mediated deletion, the frequency of the SVA recombination-mediated deletion was very low. The physical proximity and sequence similarity between repetitive elements are important factors in causing recombination between them. Therefore, the low density of SVA elements in the human genome and high sequence diversity among their VNTR regions probably resulted in the low frequency of the SVA recombination-mediated deletion in the human genome.

### 3.3. Characterization of SRADs

Seven different SVA subfamilies (SVA_A to SVA_F [9] and SVA2 [13]) exist, which emerged in chronological order in primate genomes. Among them, SVA2 is the oldest subfamily [13, 27]. In contrast, SVA_E and SVA_F subfamilies are human-specific and, thus, are younger than other subfamilies [9]. As shown in Table 3, we investigated subfamily of the SVA elements involved in the SRAD events by using RepeatMasker utility which is one of the most popular tools to annotate mobile elements. As for the SRADs, we expected that all of the SVA elements would belong to one of the SVA_A to SVA_D subfamilies because this study aimed to identify the SRADs mediated by the SVA elements which were inserted into the host genomes before the divergence of the human and chimpanzee lineages. However, the result showed that one event was mediated by the SVA_F element. We examined this SVA element in greater detail. The element is truncated and shared in the human, chimpanzee, gorilla, and orangutan genomes. As such, this SVA element must be inserted before the divergence of the gorilla and orangutan lineages, which means that it is not human specific. Through the pairwise sequence alignments of this SVA element and consensus sequence for each SVA subfamily, we found out that this element contains a DNA sequence which is specific to SVA2 subfamily [13]. We used RepeatMasker utility to annotate subfamily of SVA elements, but this tool does not contain the information about the SVA2 subfamily, which caused the annotation error.

The total amount of genomic sequence deleted by these SRAD events in the human genome after the divergence of human and chimpanzee was estimated to be 15,752 bp. As shown in Table 1, the SRADs ranged in size between 113 bp and 4,980 bp with an average size of 1,212 bp. After the divergence of humans and chimpanzees, *Alu* elements deleted approximately 400 kb of the human genome through the recombination between the two different *Alu* elements [7]. The previous study found a recombination hotspot (predominant recombination breakpoint position) on the *Alu* element. Thus, we examined the breakpoint positions on the SVA elements responsible for the SRADs. Five SRADs and four SRADs had the breakpoint on the *Alu*-like and SINE-R regions, respectively, but no predominant breakpoint position was observed on the elements. However, we could not rule out that this was a false negative result due to the low sample number of SRAD events. We further examined the types of repetitive sequences involved in the breakpoints at all SRAD loci, and the results are shown in Table 4. 

### 3.4. Characterization of SIMDs

We examined subfamilies of the SVA elements involved in the SIMD events and described the result in Table 3. Interestingly, the result showed that the SVA elements involved in 9 out of 13 SIMDs belong to SVA_D subfamily. SVA_D subfamily is the largest subfamily and accounts for over 40% of SVA elements existing in the human genome [9]. Thus, we suggest that the copy number of SVA elements is proportionally related to the frequency of SIMD events. We further characterized SIMDs focusing on their deletion size. The result showed that the deletion size ranges from 14 to 8,741 bp deleting a total of 30,785 bp of human genomic sequence since the divergence of human and chimpanzee. One of the previous studies about L1 element examined L1 insertion-mediated deletions in the human genome and reported the nonrandom distribution in their deletion size, either very short (<100 bp) or relatively large (>1 kb) [6]. In contrast, the SIMDs were randomly distributed in size as shown in Table 2.

### 3.5. Genomic Environment of SADs

The genome-wide average GC content in the human genome is 41% [10]. SVA elements are preferentially inserted into GC-rich regions in the human genome. In particular, the distribution of younger SVA elements (<5 Myrs) showed a peak at 48–50% GC content [9]. It is known that the GC-rich regions have higher gene densities [28]. As such, human genomic deletions that occur in the GC-rich regions would have increased chances to influence gene expression and cause genomic instability. We investigated the GC content in the flanking regions of SRAD and SIMD events. The GC contents in their flanking regions averaged 42.5% and 42.6%, respectively. One sample *t*-test showed that these two estimates were not significantly higher than the genome-wide GC content. We further examined whether the SAD loci resided on the intergenic region or intragenic region. The results showed that five SRAD and seven SIMD events occurred in the intragenic region of the human genome. Among the seven SIMD events, one deleted two exons in a human gene, but none of SRADs involved exons. An SRAD event that is deleterious to the host genome would have a very low probability of becoming fixed in the genome. However, this does not mean that the SRAD events are unable to cause human disease or genomic instability. In fact, neurofibromatosis, a genetic disorder of the nervous system, was caused by SRAD events in humans; an SVA element existing in intron 4 of the human neurofibromatosis type 2 (NF2) gene caused intragenic deletions in the genes of two patients with neurofibromatosis [29].

### 3.6. SIMD as an Agent Affecting Human-Chimpanzee Divergence

One of previous studies about transcriptional structures of chimpanzee sperm development-associated genes reported that the transcript of chimpanzee *tMDC II* (metalloproteinase-like, disintegrin-like and cysteine-rich domain) gene has different structure compared with that of human [30]. In addition, another study found out that human *tMDC II* transcripts are nonfunctional due to exon deletion in the gene [31]. Nonetheless, the mechanism responsible for the exon deletion has not been studied. In this study, we discovered that SIMD caused the loss of two exons in human *tMDC II* gene compared with its orthologous chimpanzee gene. *tMDC II* gene is a member of the *tMDC* gene family which plays a role in sperm-egg binding prior to fertilization [32]. As such, we suggest that the process of SIMD could be a factor causing the divergence of humans and chimpanzees, contributing to the reduction in fertilizing ability in humans. In addition, the SIMDs happened in the intragenic or even intergenic regions may also influence the levels of human gene expression through the alteration of gene regulatory regions or gene splicing patterns which could result in genetic or phenotypic difference between humans and chimpanzees.

## 4. Conclusion

Since the release of the human and chimpanzee genome reference sequences, various mechanisms responsible for the magnitude of retrotransposon-mediated genomic rearrangements have been proposed. In this study, we identified SRAD and SIMD events in the human genome through a genome-wide analysis. Our results showed that the events have deleted ~46.5 kb of human genomic sequence, since the divergence of human and chimpanzee lineages. Among the deletions, one SIMD is responsible for non-functional human *tMDC II* gene by causing a loss of two exons in the gene. Through those mechanisms, all SVA elements existing in the human genome may have the potential to cause genomic deletions that could result in human disease. This study is the first whole genome analysis to investigate human genomic deletions generated by SRAD and SIMD events and suggests the possibility that those SVA elements play a role in lineage-specific changes in the human and other hominid genomes.

## Supplementary Material

The sequences of the oligonucleotide primers, annealing temperatures, and PCR product sizes are summarized.

## Figures and Tables

**Table 1:** Characterization of the SVA recombination-associated deletion loci.

Locus	Genomic position (hg19)	Deletion size (bp)	Deletion mechanism^a^	Microhomology (bp)
87	chr1: 35413213–35414369	1017	NHEJ	4
273	chr1: 181923989–181924621	682	NHEJ	0
361	chr1: 249205332–249205990	4980	NHEJ	5
432	chr2: 44288699–44288879	277	NHEJ	7
574	chr2: 184655950–184658043	113	NHEJ	2
689	chr3: 42024110–42025361	4463	NHEJ	2
1505	chr7: 55378897–55379920	1271	NHEJ	0
1652	chr8: 11186832–11188441	305	NHEJ	3
2299	chr11: 3418351–3419320	367	NHEJ	2
2335	chr11: 46606606–46606899	526	NHEJ	2
2493	chr12: 18672855–18672949	589	NAHR	36
2836	chr15: 20408545–20409855	312	NHEJ	4
2981	chr16: 30170002–30170483	850	NHEJ	1

^a^NHEJ: nonhomologous end joining, NAHR: nonallelic homologous recombination.

**Table 2:** Location and deletion size of the SVA insertion-mediated deletion loci.

Locus	Genomic position (hg19)	Deletion size (bp)
242	chr3: 14909866–149099196	2581
732	chr3: 61656812–61658447	19
831	chr12: 31333177–31333788	347
1547	chr7: 75581297–75582629	653
1667	chr22: 35021272–35022965	1633
1671	chr14: 84565214–84566566	8741
1755	chr8: 122668707–122669486	1191
2263	chr8: 57983370–57984835	14
2508	chr8: 145092008–145092734	4859
2809	chr8: 34951832–34952650	2791
2817	chr1: 160905975–160906748	1929
3019	chr10: 101851975–101854321	5997
3577	chr16: 67746159–67746860	30

**Table 3:** SVA subfamilies involved in SVA-associated events.

SVA subfamilies	The number of SRADs	The number of SIMDs
A	2	0
B	3	1
C	3	2
D	3	11
E	0	1
F	0	0
SVA2	1	0

**Table 4:** Types of repetitive sequences involved in the breakpoints of SRAD loci.

Locus	Left break point	Right break point
87	Unique sequence	SVA_B^(1)^
273	L1MA5A	SVA_B^(2)^
361	SVA_B^(2)^	Unique
432	SVA2^(6)^	Unique
574	SVA_A^(1)^	L1PA5
689	SVA_C^(1)^	ERVL-E-int
1505	SVA_D^(2)^	Unique sequence
1652	*Alu*Jr4	SVA_C^(5)^
2299	Simple repeat (TA)	SVA_D^(1)^
2335	*Alu*Sx1	SVA_D^(3)^
2493	SVA_A^(4)^	SVA_A^(4)^
2836	SVA_C^(1)^	Unique
2981	SVA_A^(2)^	*Alu*Sg4

Each number in the parenthesis indicates the localization of the break point in the SVA element (^(1)^: *Alu*-like, ^(2)^: VNTR, ^(3)^: SINE-R, ^(4)^: poly-A tail, ^(5)^: hexamer repeats, and ^(6)^: DNA sequence specific to SVA2.)
